# Preference for daily versus on‐demand pre‐exposure prophylaxis for HIV and correlates among men who have sex with men: the China Real‐world Oral PrEP Demonstration study

**DOI:** 10.1002/jia2.25667

**Published:** 2021-02-15

**Authors:** Jing Zhang, Jun‐Jie Xu, Hong‐Yi Wang, Xiao‐Jie Huang, Yao‐Kai Chen, Hui Wang, Zhen‐Xing Chu, Qing‐Hai Hu, Xiao‐Qing He, Yao Li, Lu‐Kun Zhang, Zhi‐Li Hu, Ran‐Tong Bao, Shang‐Cao Li, Hang Li, Hai‐Bo Ding, Yong‐Jun Jiang, Wen‐Qing Geng, Sean Sylvia, Hong Shang, the CROPrEP Group

**Affiliations:** ^1^ NHC Key Laboratory of AIDS Immunology (China Medical University) National Clinical Research Center for Laboratory Medicine The First Affiliated Hospital of China Medical University Shenyang China; ^2^ Key Laboratory of AIDS Immunology Chinese Academy of Medical Sciences Shenyang China; ^3^ Key Laboratory of AIDS Immunology of Liaoning Province Shenyang China; ^4^ Collaborative Innovation Center for Diagnosis and Treatment of Infectious Diseases Hangzhou China; ^5^ Peking Union Medical College Hospital Chinese Academy of Medical Sciences Beijing China; ^6^ Beijing Youan Hospital Capital Medical University Beijing China; ^7^ Chongqing Public Health Medical Center Chongqing China; ^8^ Department of Infectious Diseases Shenzhen Third People’s Hospital Shenzhen China; ^9^ Gillings School of Global Public Health University of North Carolina at Chapel Hill Chapel Hill NC USA

**Keywords:** HIV, pre‐exposure prophylaxis, men who have sex with men, preference, regimen, switch

## Abstract

**Introduction:**

This study explores the preference for daily versus on‐demand pre‐exposure prophylaxis (PrEP) among men who have sex with men (MSM) in developing countries when both regimens are available.

**Methods:**

From 11 December 2018 to 19 October 2019, we recruited MSM for an open‐label real‐world PrEP demonstration study in four major cities in China. Subjects selected their preferred PrEP (oral tenofovir/emtricitabine) regimen (daily vs. on‐demand) at recruitment and underwent on‐site screening before initiation of PrEP. We used logistic regression to assess preference for daily PrEP and correlates.

**Results:**

Of 1933 recruited MSM, the median age was 29 years, 7.6% was currently married to or living with a female; the median number of male sexual partners was four and 6.1% had used post‐exposure prophylaxis (PEP) in the previous six months. HIV infection risk was subjectively determined as very high (>75%) in 7.0% of subjects, high (50% to 75%) in 13.3%, moderate (25% to 49%) in 31.5% and low or none (0% to 24%) in 48.1%. On average, participants preferred on‐demand PrEP over daily PrEP (1104 (57.1%) versus 829 (42.9%)) at recruitment. In multivariable analysis, currently being married to or living with a female was associated with 14.6 percentage points lower preference for daily PrEP (marginal effect = −0.146 [95% CI: −0.230, −0.062], *p* = 0.001); whereas the number of male sexual partners (marginal effect = 0.003 [95% CI: 0.000, 0.005], *p* = 0.034) and a subjective assessment of being very high risk of HIV infection (vs. low and no risk, marginal effect size = 0.105 [95% CI: 0.012, 0.198], *p* = 0.027) were associated with increased preference for daily versus on‐demand PrEP. Among the 1933 potential participants, 721 (37.3%) did not attend the subsequent on‐site screening. Lower‐income, lower education level, lower subjective expected risk of HIV infection risk and younger age positively correlated with the absence of on‐site screening.

**Conclusions:**

MSM in China prefer both daily and on‐demand PrEP when both regimens are provided free. Social structural factors and subjective risk of HIV infection have significant impacts on PrEP preference and use. The upcoming national PrEP guideline should consider incorporating both regimens and the correlates to help implement PrEP in China.

## 1 INTRODUCTION

Daily oral pre‐exposure prophylaxis (PrEP) for human immunodeficiency virus (HIV) with tenofovir disoproxil fumarate plus emtricitabine (TDF/FTC) reduced the risk of HIV‐1 infection among men who have sex with men (MSM) in a blinded randomized controlled trial [1, 2] and a pragmatic open‐label randomized trial [3]. In 2012, the US Food and Drug Administration approved oral PrEP for individuals at substantial risk of HIV infection, irrespective of sexual orientation or behaviour. However, the high pill burden and low rates of adoption and adherence to a daily PrEP regimen stimulated investigations into other modalities of PrEP, including an on‐demand regimen that requires fewer pills than daily PrEP [4]. Evidence from randomized controlled trials since 2015 showed that on‐demand PrEP was associated with an 86% reduction in HIV‐1 infection rate compared to placebo among MSM [4] and was highly effective in an open‐label extension [5, 6]. In 2019, the World Health Organization incorporated on‐demand PrEP guidelines for MSM [7].

Because of the recommendations for both daily and on‐demand PrEP for MSM, four trials were conducted in developed countries to determine preferences for one regimen over the other and the underlying factors among MSM [8, 9, 10]; this was particularly important because daily and on‐demand regimens differ in terms of pill burden, adherence and cost‐effectiveness [11, 12, 13]. Studies in Belgium, Australia and the Netherlands reported that 66% to 76.5% of MSM preferred daily PrEP, whereas in France, where on‐demand PrEP has been recommended since 2015, around 60% of MSM chose on‐demand PrEP in the first year of the trial [14]. Daily PrEP was more often the preference of those who perceive a higher risk of HIV infection [8, 10], have condomless anal intercourse (CLAI) at a higher frequency, and have more sexual partners [6]. On‐demand PrEP was the more common choice when the risk of HIV infection was predictable, and sex was less frequent [8, 10, 15]. These findings suggested that varying levels of awareness of PrEP regimens, HIV‐related behaviour and the subjectively perceived risk of HIV infection influence preferences for particular PrEP regimens.

Data regarding preferences for PrEP regimens among MSM in low‐ and middle‐income countries (LMICs) are scarce, although these countries shoulder the most substantial burden of the epidemic. LMICs harbour more than three‐quarters of people living with HIV, and they continue to be the hardest hit by new infections, especially among key populations such as MSM [16]. High levels of stigmatization of homosexuality [17], high occurrence of HIV‐related risk behaviour [18], low subjective expected risk of HIV infection [19], but much lower awareness of PrEP [20] among MSM in LMICs than their counterparts in developed countries, all pose challenges for the implementation of PrEP in LMICs.

In China, MSM accounted for 23.3% of newly reported HIV/AIDS cases in 2018 [21]. Currently, fewer than 1% of the estimated 8,226,000 MSM in China are using PrEP to prevent HIV [22]. One reason for the low take‐up of PrEP may be that homosexuality remains highly stigmatized in China: 31.2% of MSM in China have female sexual partners or are married to a woman [23]. The concealment of homosexuality places the general female population at greater risk [24] and raises barriers to their access to HIV prevention resources.

In this study, we investigated preferences for daily versus on‐demand PrEP among MSM and the factors that influence this choice as part of an ongoing open‐label real‐world demonstration study of MSM in China [25].

## 2 METHODS

### 2.1 Study design and setting

Starting on 11 December 2018, there has been an open‐label real‐world PrEP demonstration project (China Real‐world Study of Oral PrEP [CROPrEP]) in four major cities in China: Beijing, Shenyang, Chongqing and Shenzhen. This study receives support from the Action Plan for the 13^th^ 5‐Year Plan of the Mega Programme of the China National Health and Family Planning Commission. The study enrolled 1000 MSM who use oral PrEP and prospectively followed them for 12 months with quarterly visits. Both daily and on‐demand PrEP regimens were available free of charge for qualified subjects. This study aimed to systematically collect comprehensive empirical data to determine effective ways to implement PrEP among MSM in China. The protocol’s registration with the Chinese Clinical Trial Registry occurred on 8 December 2017 (no. ChiCTR‐IIN‐17013762). The protocol previously appeared in print [25].

The four study sites (Youan Hospital of Capital Medical University in Beijing, The First Affiliated Hospital of China Medical University in Shenyang, Chongqing Public Health Medical Centre of Southwest University in Chongqing and The Third People’s Hospital of Shenzhen) are tertiary hospitals that provide HIV counselling and testing and HIV treatment by physicians specializing in infectious diseases. Because of the well‐documented protective effect of PrEP, we did not perform a power calculation for the CROPrEP demonstration project at any site. We employed the largest sample size possible at each site, considering the human resources and capability at each site [25].

All four study sites worked closely with local community‐based organizations (CBOs) through recruitment, enrolment and follow‐up. The CBOs provided advice during implementation, facilitated advertising for the study in the MSM community during recruitment, and strengthened group‐ and individual‐level supervision of cohort management during follow‐up.

### 2.2 Recruitment and data collection

We recruited subjects from 11 December 2018 to 19 October 2019. Recruitment occurred both online and offline. Online recruitment included advertising on social media accounts managed by the study team and forwarded by the CBOs at each study site. The offline recruitment included voluntary counselling and testing clinics at the local centres for disease control and prevention (CDC), brochures distributions and outreach events run by the CBOs and peer referrals. Both online and offline recruitment materials provided text and multimedia links for the introduction of PrEP, the various regimens provided in the study, and a QR code for preregistration in the study. By scanning the QR code, potential participants accessed an electronic informed consent and a brief self‐administrated questionnaire. After finishing the questionnaire, there was an invitation for on‐site screening. We previously published recruitment details in the study protocol [25].

This questionnaire collected the following data: (1) demographics including age, education level, marital status, monthly income; (2) male gender at birth (yes/no); (3) unprotected (condomless) receptive anal intercourse with male partners in the previous six months; (4) number of male partners (regardless of condom use and HIV serostatus) in the previous six months; (5) history of sexually transmitted infections (STIs) in the previous six months: syphilis, HSV‐2, gonorrhoea, chlamydia, chancroid or lymphogranuloma venereum (yes/no); (6) usage of recreational drug use in the previous six months: alkyl nitrites, ecstasy, methamphetamine, amphetamine, tramadol or ketamine (yes/no); (7) history of post‐exposure prophylaxis (PEP) (yes/no); (8) subjective expected risk of HIV infection, asked as follows: “Referring to your past and present sexual practice, at which level do you rank your risk of contracting HIV?” We asked the participants to choose one from the following four options: A. “Very high, which means more than 75% of the chance of getting infected with HIV,” B. “High, which means between 50% and 75% of the chance of getting infected with HIV,” C. “Moderate, which means between 25% and 49% of the chance of getting infected with HIV” or D. “Low or none, which means between 0% and 24% of the chance of getting infected with HIV”; (9) willingness to join the study and the preference for PrEP regimens (daily/on‐demand) and (10) contact information (phone number/QQ number/WeChat number).

After finishing the questionnaire, eligible candidates preregistered and received invitations for an on‐site screening visit at the local study centre on the principle of competitive enrolment. The eligibility criteria for the invitation of on‐site screening were as follows: (1) being male gender at birth and have sex with men; (2) having an interest in using oral PrEP with daily regimen or on‐demand regimen; (3) provision of contact information; (4) provided of informed consent and (5) willingness to join the study.

### 2.3 On‐site screening and data collection

We asked eligible potential participants to attend the on‐site screening within four weeks. We identified potential participants attending the on‐site screening using the previously provided contact information. The on‐site screening included formal written, informed consent and comprehensive screening at each study site. The screening included laboratory testing for antibodies and RNA for HIV, syphilis, herpes simplex virus 2, hepatitis B, bone mineral density, renal and liver function, blood glucose and lipids, clinical evaluation with family or personal medical history, consultation of PrEP and regimens with a physician and a self‐administered questionnaire. More details of laboratory testing are available in the published protocol [25]. All participants received 50 RMB (about US $7.07) for each on‐site visit to defray travel costs and missed work.

The on‐site self‐administered questionnaire documented sexual behaviour in the previous three months, including the type of sexual partner and the number of condoms used with each type of sexual partner. We did not record any repeated information from the questionnaire at recruitment other than the willingness to join the study and the PrEP regimen preference (daily/on‐demand).

Eligible criteria for enrolment in this study were as follows: (1) age 18 to 65 years; (2) test results demonstrating HIV‐negativity; (3) male gender at birth and having sex with men; (4) having at least one of the following behaviours in the previous six months: unprotected receptive anal intercourse with a male, more than two male partners in the past six months, STIs in the previous six months, or history of PEP; (5) comprehensive screening examination without abnormal indicators; (6) ability and willingness to provide written informed consent and (7) Chinese citizenship.

### 2.4 PrEP regimens and provision

This study's oral PrEP was Truvada (emtricitabine 200 mg/tenofovir disoproxil fumarate 300 mg tablets). The medication was provided free to eligible participants after enrolment. Participants chose PrEP in a daily regimen or on‐demand regimen. The daily regimen was one pill of Truvada every 24 hours. The on‐demand regimen was two pills of Truvada at least two to twenty‐four hours before sex (or one pill if the previous dose was taken one to six days prior), one pill 24 hours after the first dose and one pill 48 hours after the first dose; if one continues to have sex beyond 24 hours after the first double dose, the participant continued to take a single dose every 24 hours until one final dose approximately 24 hours after the final sexual intercourse. After on‐site screening, eligible participants enrolled and called back to pick up the medication for PrEP initiation. Every enrolled participant received three bottles of Truvada, with 30 pills per bottle, at baseline and quarterly follow‐up at the study site. The study staff counted the pills remaining from the previous three months retracted them at each follow‐up visit.

### 2.5 Statistical analysis

We analysed data using STATA/SE v15.1 (Stata Corp., College Station, TX, USA). We recorded demographic characteristics, HIV‐related risk behaviour and subjective expected risk of HIV, as well as preference for PrEP regimens. We analysed factors correlated with daily regimen preference versus on‐demand PrEP using both univariate correlations and multivariable logistic regression. We adjusted for the study site as a covariate in the multivariable analysis. Average marginal effects are reported for logistic regressions. We compared demographic characteristics, HIV‐related behaviours and subjective expected risk of HIV between potential participants who attended the on‐site screening and those who did not use the Chi‐square test. A *p*‐value of less than 0.05 was statistically significant.

### 2.6 Ethics

The Institutional Review Boards at the four participating research centres reviewed and approved the study protocol and informed consent forms. The centres were The First Affiliated Hospital of China Medical University ([2018]2015–139‐5), Beijing Youan Hospital Capital Medical University ([2018]‐037), the Third People’s Hospital of Shenzhen (2018–007) and Chongqing Public Health Medical Centre (2018–004‐02‐KY). We informed all subjects that participation was voluntary. Their information would remain anonymous before starting the questionnaire; the participants did this by clicking a button and reading the online consent form. Separately, we obtained written informed consent from each subject before consultation with a physician before the on‐site screening. Subjects took part in the study voluntarily and had the right to refuse to answer any of the questions or withdraw from the study without penalty.

## 3 RESULTS

**Figure 1:**
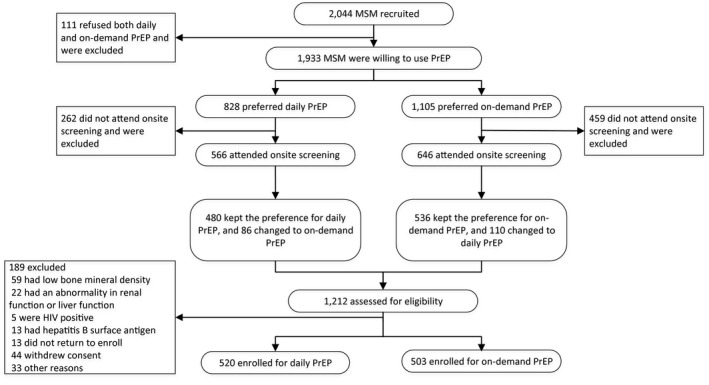
Flowchart of willingness and decision making of the participants of the PrEP demonstration study of in China, 2019

From 11 December 2018 to 19 October 2019, there were 2044 MSM who completed and submitted the online recruitment questionnaires. Among these 2044 MSM, 1,933 (94.6%, 1933/2044) showed interests in starting PrEP, and 111 (5.4%, 111/2044) refused to use oral PrEP. We invited all 1933 potential participants to the on‐site screening before initiation of PrEP. During the on‐site screening, 1,212 (62.7%, 1212/1933) attended the study sites, and we assessed them for eligibility (Figure 1).

After the on‐site screening, we enrolled those who completed all tests and without abnormal results or indicators and were called back to pick for the medication for PrEP initiation. We excluded 189 (15.6%, 189/1212) MSM, including 59 with low bone mineral density, 22 with abnormalities in renal and liver function, five with HIV‐positivity, 13 with hepatitis B surface antigen, 13 who did not return to pick up the medication, 44 who withdrew consent and 33 for other reasons. There were 520 enrolled for daily PrEP, and 503 enrolled for on‐demand PrEP (Figure 1).

### 3.1 Preference for daily and on‐demand PrEP

During recruitment, there were 828 (42.8%, 828/1933) MSM among the 1933 potential participants who showed interest in oral PrEP instead of daily PrEP. During on‐site screening, 262 (31.6%, 262/828) of those who preferred daily PrEP did not return. There were 566 MSM who preferred daily PrEP, attended the on‐site screening, and provided informed consent, among which 86 (15.2%, 86/566) MSM changed their preference from daily PrEP to on‐demand PrEP after consultation with a physician during on‐site screening; 480 MSM maintained their preference for daily PrEP.

During the recruitment, there were 1,105 (57.2%, 1105/1933) potential participants who preferred on‐demand PrEP. During on‐site screening, 459 (41.5%, 459/1105) of those who preferred on‐demand PrEP did not return. There were 646 MSM who preferred on‐demand PrEP, attended the on‐site screening, and provided informed consent, among which 110 (17.0%, 110/646) changed their preference from on‐demand PrEP to daily PrEP after consultation with a physician during the on‐site screening; 536 MSM maintained their preference for on‐demand PrEP.

### 3.2 Demographic information and HIV‐related risk behaviours of the study population

The median age was 29.0 years old (25% to 75% quartiles, 25.0, 36.0). The majority had education levels of undergraduate or higher (76.7%, 1482/1933), monthly income <6000 CNY (857 USD) (58.2%, 1125/1933) and were currently not married (92.4%, 1786/1933). Regarding HIV‐related risk behaviours in the previous 6 months, the median number of male sexual partners was 4.0 (25% to 75% quartiles, 2.0, 6.0); 6.1% (117/1933) had used PEP, 63.4% (1226/1933) had engaged in CLAI, 12.4% (240/1933) had at least one STI and 41.0% (793/1933) had used recreational drugs. When questioned on subjective expected risk of HIV infection, 7.0% (136/1933) of subjects reported very high risk (>75%); 13.3% (258/1933) reported being at high risk (50% to 75%), 31.5% (609/1933) reported moderate risk (25% to 49%) and 48.1% (930/1933) reported low or no risk (0% to 24%) (Table 1).

**Table 1:** Demographics, HIV related behaviour and subjective expected HIV infection risk of MSM who have preference for daily and on‐demand PrEP in China, 2019 (n = 1933)

Characteristics	Total (N/%)	Daily PrEP (N/%)	On‐demand PrEP (N/%)
Total	1933 (100.0)	828 (42.8)	1105 (57.2)
Age (years) [Median (quantiles)]	29.0 (25.0, 36.0)	29.0 (25.0, 35.0)	30.0 (25.0, 36.0)
Education level			
High school or below	451 (23.3)	172 (20.8)	279 (25.2)
Undergraduate and above	1482 (76.7)	656 (79.2)	826 (74.8)
Monthly income (USD)			
0 to 857	1125 (58.2)	469 (56.6)	656 (59.4)
≥858	808 (41.8)	359 (43.4)	449 (40.6)
Marital status			
Currently married with female	147 (7.6)	40 (4.8)	107 (9.7)
Not married (Single, in a relationship or habitat with male, separated, divorced with female or widowed)	1786 (92.4)	788 (95.2)	998 (90.3)
Have been on Post‐exposure Prophylaxis in the previous six months			
Yes	117 (6.1)	61 (7.4)	56 (5.1)
No	1816 (93.9)	767 (92.6)	1049 (94.9)
Number of male sexual partners in the previous six months (Median, quantiles)	4.0 (2.0, 6.0)	4.0 (3.0, 6.0)	3.0 (2.0, 5.0)
Had CLAI in the past six months			
Yes	1226 (63.4)	533 (64.4)	693 (62.7)
No	707 (36.6)	295 (35.6)	412 (37.3)
Have diagnosis of any STIs in the previous six months^a^			
Yes	240 (12.4)	116 (14.0)	124 (11.2)
No or don’t know	1693 (87.6)	712 (86.0)	981 (88.8)
Recreational drug usage in the previous six months^b^			
Yes	793 (41.0)	362 (43.7)	431 (39.0)
No	1140 (59.0)	466 (56.3)	674 (61.0)
Subjective expected risk of HIV infection of self (as in 0% to 100%)			
Very high risk (>75%)	136 (7.0)	71 (8.6)	65 (5.9)
High risk (75% to 50%)	258 (13.3)	133 (16.1)	125 (11.3)
Median risk (49% to 25%)	609 (31.5)	258 (31.2)	351 (31.8)
Low and no risk (24% to 0%)	930 (48.1)	366 (44.2)	564 (51.0)

CLAI, condomless anal intercourse; MSM, men who have sex with men; PrEP, pre‐exposure prophylaxis; STIs, sexually transmitted infections.

^a^ STIs included syphilis, HSV‐2, gonorrhoea, chlamydia, chancroid, lymphogranuloma venereum and others

^b^ recreational drugs included alkyl nitrites, ecstasy, methamphetamine, amphetamine, tramadol, ketamine and others.

### 3.3 Correlates of PrEP regimen preference

Compared to MSM who preferred on‐demand PrEP, those who preferred the daily regimen were more likely to have education levels of undergraduate or higher (79.2% vs. 74.8%), were more likely not to be currently married to or living with a woman (4.8% vs. 9.7%), and had a higher‐than‐moderate subjective risk of HIV infection (very high risk, 8.6% vs. 5.9%; high risk, 16.1% vs. 11.3%) (Table 1).

The univariate analysis revealed that having an education level of undergraduate or above (vs. high school and below) correlated with a 6.2%point increase in preference for daily PrEP (marginal effect = 0.062 [95% CI: 0.001, 0.114], *p* = 0.021). Being currently married to or living a woman correlated with a 18.2 percentage point decrease in preference for daily‐PrEP (marginal effect = −0.182 [95% CI: −0.271, −0.092], *p* < 0.001). Each additional male sexual partner in the previous six months was associated with a 0.4 percentage point increase in preference for daily‐PrEP (marginal effect = 0.004 [95% CI: 0.001, 0.007], *p* = 0.004). Having a high and very high risk of HIV infection (vs. no and low risk) correlated with a 12.2 percentage point (marginal effect = 0.122 [95% CI: 0.053, 0.191], *p* < 0.001) and 12.9 percentage point (marginal effect = 0.129 [95% CI: 0.038, 0.218], *p* = 0.005) increase in preference for daily PrEP respectively.

After adjustment for study site, we entered correlates with *p*‐values less than 0.2 in the univariable analysis into the multivariable analysis. We found that being currently married to or living with a woman correlated with a 14.6 percentage point decrease in preference for daily‐PrEP (marginal effect = −0.146 [95% CI: −0.230, −0.062], *p* = 0.001). Each additional male sexual partner correlated with a 0.3 percentage point increase in preference for daily‐PrEP (marginal effect = 0.003 [95% CI: 0.000, 0.005], *p* = 0.034). High and very high subjective risk of HIV infection (vs. no and low risk) correlated with a 11.5 percentage point (marginal effect = 0.115 [95% CI: 0.045, 0.185], *p* = 0.001) and 10.5 percentage point (marginal effect = 0.105 [95% CI: 0.012, 0.198], *p* = 0.027) increase in preference for daily PrEP respectively (Table 2).

**Table 2:** Univariable and multivariable marginal effect analysis of correlates of preference for daily‐PrEP over on‐demand PrEP among MSM in China, 2019 (N = 1933)

Variables	Marginal effect (95% CI)	*p*‐value	Adjusted marginal effect (95% CI)^a^, ^b^	*p*‐value
Age (years)	−0.002 (−0.005, 0.000)	0.074	−0.001 (−0.004, 0.002)	0.395
Education level be undergraduate and above	0.062 (0.001, 0.114)	0.021	0.044 (−0.012, 0.100)	0.122
Monthly income >857 (USD)	0.027 (−0.017, 0.072)	0.229		
Married or living with female	−0.182 (−0.271, −0.092)	<0.001	−0.146 (−0.230, −0.062)	0.001
Have been on Post‐exposure Prophylaxis in the past six months	0.097 (0.006, 0.188)	0.036	0.067 (−0.028, 0.161)	0.167
Number of male sexual partners in the past six months	0.004 (0.001, 0.007)	0.004	0.003 (0.000, 0.005)	0.034
Had CLAI in the past six months	0.018 (−0.028, 0.063)	0.454		
Received diagnosis of any STIs in the past six months^c^	0.062 (−0.004, 0.128)	0.065	0.054 (−0.014, 0.122)	0.122
Recreational drug usage in the past six months	0.048 (0.004, 0.093)	0.034	0.019 (−0.027, 0.066)	0.412
Subjective risk of HIV infection (as in 0% to 100%)^d^, ^e^				
Very high risk (100% to 76%)	0.129 (0.038, 0.218)	0.005	0.105 (0.012, 0.198)	0.027
High risk (75% to 50%)	0.122 (0.053, 0.191)	<0.001	0.115 (0.045, 0.185)	0.001
Median risk (49% to 25%)	0.030 (−0.020, 0.080)	0.241	0.015 (−0.036, 0.066)	0.564

MSM, men who have sex with men; PrEP, pre‐exposure prophylaxis; STIs, sexually transmitted infections.

^a^ The study site was adjusted

^b^ variables with a *p* < 0.2 in univariable analysis were included in the multivariable model

^c^ STIs included syphilis, HSV‐2, gonorrhoea, chlamydia, chancroid, lymphogranuloma venereum and others

^d^ compared to “low and no risk (0% to 24%)”

^e^ recreational drugs included alkyl nitrites, ecstasy, methamphetamine, amphetamine, tramadol, ketamine and others.

### 3.4 Comparison between potential participants who attended and did not attend on‐site screening

Among all 1,933 potential participants reporting interest in using oral PrEP during recruitment, 721 (37.3%) did not attend the subsequent on‐site screening. Compared to the MSM who attended the on‐site screening, those who did not attend were more likely to have a preference for on‐demand PrEP (63.7% vs. 53.3%, *p* < 0.001), to be younger (median 28‐year‐old vs. 30‐year‐old, *p* < 0.001), to have an education level of high school or below (28.3% vs. 20.4%, *p* < 0.001), to have monthly income of $0 to 857 USD (67.0% vs. 53.0%, *p* < 0.001), to have fewer male sexual partners in the previous six months (median 3 vs. 4, *p* < 0.001) and to have subjectively expected low and no risk of HIV infection (51.7% vs. 46.0%, *p* = 0.049). Compared to the MSM who attended the on‐site screening, those who did not attend were less likely to engage in CLAI in the previous six months (60.3% vs. 65.3%, *p* = 0.029) or use a recreational drug in the previous six months (37.4% vs. 43.2%, *p* = 0.014) (Table 3).

**Table 3:** Comparison between MSM who attended the onsite screening and those who did not among MSM who had a preference of PrEP in China, 2019 (n = 1933)

Characteristics	Total (N/%)	MSM who attended the onsite screening (N/%)	MSM who did not attend the onsite screening (N/%)	χ^2^	*p*‐value
Total	1933 (100.0)	1212 (62.7)	721 (37.3)		
Preference of PrEP					
Daily PrEP	828 (42.8)	566 (46.7)	262 (36.3)	19.820	<0.001
On‐demand PrEP	1105 (57.2)	646 (53.3)	459 (63.7)		
Age (years) (mean, quantiles)	29.0 (25.0, 36.0)	30.0 (25.0, 36.0)	28.0 (24.0, 35.0)		<0.001
Education level					
High school or below	451 (23.3)	247 (20.4)	204 (28.3)	15.830	<0.001
Graduate and above	1482 (76.7)	965 (79.6)	517 (71.7)		
Monthly income (USD)					
0 to 857	1125 (58.2)	642 (53.0)	483 (67.0)	36.526	<0.001
≥858	808 (41.8)	570 (47.0)	238 (33.0)		
Marital status					
Married with female	147 (7.6)	85 (7.0)	62 (8.6)	1.618	0.203
Single, in a relationship or habitat with male, separated, divorced with female or widowed	1786 (92.4)	1127 (93.0)	659 (91.4)		
Have you been on Post‐exposure Prophylaxis in the past six months?					
Yes	117 (6.1)	72 (5.9)	45 (6.2)	0.072	0.789
No	1816 (93.9)	1140 (94.1)	676 (93.8)		
Number of male sexual partners in the past six months (mean, quantiles)	4.0 (2.0, 6.0)	4.0 (3.0, 6.0)	3.0 (2.0, 5.0)		<0.001
Had CLAI in the past six months					
Yes	1226 (63.4)	791 (65.3)	435 (60.3)	4.739	0.029
No	707 (36.6)	421 (34.7)	286 (39.7)		
Have diagnosis of any STIs in the past six months^a^					
Yes	240 (12.4)	159 (13.1)	81 (11.2)	1.476	0.224
No or don’t know	1693 (87.6)	1053 (86.9)	640 (88.8)		
Recreational drug usage in the past six months^b^					
Yes	793 (41.0)	523 (43.2)	270 (37.4)	6.079	0.014
No	1140 (59.0)	689 (56.8)	451 (62.6)		
Subjective expected risk of HIV infection of self (as in 0% to 100%)					
Very high risk (> 75%)	136 (7.0)	85 (7.0)	51 (7.1)	7.857	0.049
High risk (75% to 50%)	258 (13.3)	177 (14.6)	81 (11.2)		
Median risk (49% to 25%)	609 (31.5)	393 (32.4)	216 (30.0)		
Low and no risk (0% to 24%)	930 (48.1)	557 (46.0)	373 (51.7)		

CLAI, condomless anal intercourse; MSM, men who have sex with men; PrEP, pre‐exposure prophylaxis; STIs, sexually transmitted infections.

^a^ STIs included syphilis, HSV‐2, gonorrhoea, chlamydia, chancroid, lymphogranuloma venereum and others

^b^ recreational drugs included alkyl nitrites, ecstasy, methamphetamine, amphetamine, tramadol, ketamine and others.

## 4 DISCUSSION

To our best knowledge, this is the first study investigating the preference for daily versus on‐demand PrEP, and the associated factors thereof, among MSM in a real‐world setting in a developing country. Overall, MSM in China preferred both daily and on‐demand PrEP. Preference for daily PrEP increased with the number of male sexual partners in the previous six months and higher subjective risk of HIV infection. That preference declined with currently being married to or living with a woman. Attendance for on‐site screening was higher among MSM with a preference for the daily than on‐demand PrEP (68.3% vs. 58.5%). MSM with lower levels of education and income, with fewer male sexual partners, and lower subjective expected risk of HIV infection were more likely to fail to attend on‐site screening.

On‐demand PrEP was the preference of more MSM participants during online recruitment. There were more MSM who attended the on‐site screening preferring on‐demand PrEP. However, compared to MSM who preferred daily PrEP, a higher proportion of MSM who preferred on‐demand PrEP during the online recruitment failed to attend on‐site screening. Our finding of the higher preference for on‐demand PrEP contradicts findings of studies from Belgium, the Netherlands, Australia and North America [8, 9, 10, 26, 27]; however, our finding accords with other findings from North America [28, 29] and France [14]. It is important to note that the higher preference for daily reported in previous real‐world studies (66% to 76.5%) (providing both daily and on‐demand regimens of PrEP [8, 9, 10]) occurred among MSM who attended on‐site screening. Necessary and sufficient information regarding PrEP was provided to these MSM before recording their preferences. The two studies reporting a higher preference for on‐demand PrEP (61.99% to 75.8%) [28, 29] collected these preferences via online surveys. We assume that these preferences for on‐demand PrEP were collected before more detailed discussions with physicians. Only one study was from France, where the on‐demand PrEP regimen has been available since 2015 [6]. The authors reported a higher preference and usage of on‐demand PrEP among MSM who initiated PrEP [14]. There are three explanations for the different preferences for daily and on‐demand PrEP among MSM reported online versus on‐site: (1) MSM who chose on‐demand had lower HIV infection risk (e.g. lower number of sexual partners, less CLAI) or lower perceived HIV infection risk (e.g. lower subjective expectation of HIV infection risk), as we observed in this study; therefore, they were more reluctant to attend the on‐site screening; (2) There are many structural barriers for HIV‐risk populations to attend the on‐site screening (e.g. younger age, lower‐income), as we observed in this study. MSM who did not attend the on‐site screening were a different subpopulation from those who did and (3) Online recruitment could only provide brief information; whereas, at the on‐site visit, the participant can discuss actual risks and concerns in more detail. Hence, by presenting willingness and decision making flow regarding preference for PrEP, our study sheds light on differences in preference among the target population regarding when and how the data were collected.

To better understand the preference for PrEP regimen among MSM in China, we need to understand that the level of awareness of PrEP among MSM in China differs significantly from those of North America and France because PrEP has been available for many years in these countries. Moreover, more than 80% of MSM in the United States reported PrEP awareness in 2017 [30], whereas only 22.4% of MSM in China was aware of PrEP in that year [19]. Almost 70% of MSM in China had concerns about the side effects of PrEP [19]. Depending on how often it is taken, on‐demand PrEP could use far fewer than half the number of pills for a daily regimen over a month. Recent studies in Belgium and Netherlands found that 19% to 30% of PrEP users switched their regimen during follow‐up [5, 31], suggesting that switching regimen is relatively common and that daily and on‐demand PrEP regimens are fluid [31]. Instead of making daily and on‐demand PrEP into two different regimens, it is more reasonable to tailor PrEP use basing on one’s current risk or create a new “combined” dosing regimen. Basing on the evidence we collected in the present, we recommend providing both regimens and relevant information to MSM in China to clarify the appropriateness of different regimens in individual, real‐life settings so that MSM can make well‐informed decisions.

Marital status significantly correlated with PrEP regimen choice among MSM. Specifically, those currently married to or living with a woman were less likely to choose daily PrEP by 14.6 percentage points. This correlation may be explained by the fact that due to the high degree of stigmatization of homosexuality [32] (common in many developing countries [17]), 32% of MSM in China is married to females [23] and have never disclosed their sexual orientation to their spouses. One study reported that only 4% of MSM in China fully disclosed their sexual orientation [33]. The need to conceal status is a potential barrier to taking PrEP daily, whereas on‐demand PrEP requires less frequent pill intake, making it more easily concealable. However, according to a previous MSM study in China, MSM with bisexual behaviour have higher HIV prevalence than MSM who only have homosexual behaviour [24]. Having a female partner may, therefore, also indicate a higher risk. In countries with high stigmatization of homosexuality, clinicians should consider this conflict between high HIV infection risk and the barriers to choosing daily PrEP among married MSM when providing PrEP.

Subjective HIV infection risk predicts PrEP use and preference [20, 34]. We also found that subjective HIV infection risk correlated with access to PrEP healthcare and a 10‐percentage point increase in preference for daily over on‐demand PrEP. Because daily PrEP provides higher coverage for risky sexual behaviour, it is reasonable for MSM who have higher subjective HIV risk to choose daily‐PrEP. However, previous studies among PrEP‐eligible Chinese MSM with no PrEP experience found that subjective HIV risk was not associated with an individual’s perception of PrEP candidacy (i.e., whether or not they think they are a good candidate for PrEP) nor their intention to use PrEP (as measured using the Motivational PrEP Cascade [35]). However, men were more likely to see themselves as appropriate candidates for PrEP or willing to use PrEP if they perceived higher benefits of PrEP [36, 37]. The authors then called for the use of a “gain‐framed” approach to promote PrEP that highlights the potential benefits of PrEP among potential users instead of emphasizing individual HIV risk. We found that subjective HIV risk associated with preference for daily PrEP, a different finding from those of the studies mentioned earlier. Given that little research has been done to understand the acceptability, appropriateness and effectiveness of “gain‐framed” messaging versus “loss‐framed” messaging in the PrEP campaign among the MSM population in the Chinese context, more studies are needed to assess both approaches and to determine what works best for Chinese MSM.

We found that the number of male sexual partners was a significant individual‐level correlate of PrEP choice. In the past six months, each additional male sexual partner increased preference for daily PrEP by 6.4 percentage points. Because HIV infection strongly correlated with the number of sexual partners [38, 39], this driver demonstrated a promising impact of PrEP on HIV epidemic control among MSM; those with a higher risk of HIV infection would be protected by higher coverage of PrEP through their preference for daily PrEP. More importantly, this individual‐level benefit of PrEP users would be multiplied through their sexual network [40, 41], meaning that lowering their HIV infection risk could decrease the HIV infection risk in their entire sexual network. Future studies should measure the impact of preference of PrEP users on the sexual network of MSM to understand the effect of PrEP better.

Notably, almost 40% of the potential participants who was aware of PrEP and showed a willingness to use it did not attend the on‐site screening. This absence was a large gap in the PrEP continuum, consisting of at‐risk MSM, awareness, willingness to take PrEP, access to healthcare, receiving a prescription and adhering to effective PrEP [42]. Younger MSM, those with lower education levels, and lower monthly incomes were more likely to miss the on‐site screening, even though both the medication and testing were provided free. These factors were social structural barriers to PrEP use in many countries and settings [43, 44]. This correlation suggests that potential PrEP users who are younger and from lower‐income groups face barriers beyond financial ones. MSM with low and no subjective expected HIV infection risk were also more likely to miss on‐site screening. However, these MSM were at risk of HIV (e.g. they reported engaging in CLAI, had diagnoses of STIs, and had more than two male sexual partners in the previous six months). While more than 60% engaged in CLAI in the previous six months, more than half considered themselves to be at low and no risk of HIV infection. Despite being at high risk based on objective criteria, the perceived risk of HIV was low among MSM in China [19]. It is essential to communicate the HIV infection risk using objective risk assessment tools [45] to enable eligible PrEP users to make better choices regarding PrEP.

This study has several significant limitations. First, the participants who responded to the recruitment and attended the on‐site screening were a self‐selected sample mainly residing in four cities. A large number of individuals showed interested in PrEP but did not attend the on‐site screening. Most of the characteristics mentioned are based on the larger group instead of the MSM who initiated PrEP. Second, we were only able to assess the correlates of PrEP choice rather than the causal effects of factors driving regimen preference. Finally, our data were cross‐sectional, and we were unable to observe how PrEP choice was associated with adherence and health outcomes. Future studies should assess these causal drivers of PrEP choice and the effects of alternative approaches to offering PrEP options on adherence and health outcomes over time.

## 5 CONCLUSIONS

On average, MSM in China prefer both daily and on‐demand PrEP when both regimens are provided free. Social structural factors and subjective risk of HIV infection have significant impacts on PrEP preference and uptake. The upcoming national PrEP guidelines should consider incorporating both regimens and correlates with help implement PrEP in China.

## COMPETING INTERESTS

The authors declare that there are no conflicts of interest concerning this work.

## AUTHORS’ CONTRIBUTIONS

JZ, JJX and HS involved in conceived and designed the study. JZ, JJX, SS, HYW, XJH, YKC, HW, ZXC, QHH, XQH, YL, LKZ, ZLH, RTB, SCL, HL, HBD, YJJ and WQG performed the study and experiments. JZ, JJX and SS. analysed the data and drafted the study report. All authors reviewed and approved the final report.

### ABBREVIATIONS

CLAI, condomless anal intercourse; CBO, community‐based organization; HIV, human immunodeficiency virus; MSM, men who have sex with men; PEP, post‐exposure prophylaxis; PrEP, pre‐exposure prophylaxis; STI, sexually transmitted infection; TDF/FTC, tenofovir disoproxil fumarate plus emtricitabine.
