# CORRIGENDUM

**DOI:** 10.1002/jia2.25754

**Published:** 2021-06-12

In the article [1], there were errors in the author affiliation and correspondence information on e25667.

1. The affiliations of Xiao‐Jie Huang are incorrect and should have been changed to Center for Infectious Diseases, Beijing YouAn Hospital, Capital Medical University, Beijing, China
2. The main corresponding author for this article should be Hong Shang. Correspondence address: NHC Key Laboratory of AIDS Immunology (China Medical University), National Clinical Research Center for Laboratory Medicine, The First Affiliated Hospital of China Medical University, Shenyang, China (hongshang100@hotmail.com)
